# Parasite dynamics in the peripheral blood and the placenta during pregnancy-associated malaria infection

**DOI:** 10.1186/s12936-016-1541-x

**Published:** 2016-09-21

**Authors:** Lauren M. Cohee, Linda Kalilani-Phiri, Patricia Mawindo, Sudhaunshu Joshi, Matthew Adams, Leo Kenefic, Christopher G. Jacob, Terrie E. Taylor, Miriam K. Laufer

**Affiliations:** 1 Institute for Global Health, University of Maryland School of Medicine, Baltimore, MD USA; 2 Department of Medicine, University of Malawi College of Medicine, Blantyre, Malawi; 3 Blantyre Malaria Project, University of Malawi College of Medicine, Blantyre, Malawi; 4 Department of Osteopathic Medical Specialties, College of Osteopathic Medicine, Michigan State University, East Lansing, MI USA

**Keywords:** Malaria in pregnancy, Placental malaria, Molecular epidemiology, Genotyping, Microsatellite markers, Submicroscopic, Sulfadoxine–pyrimethamine, Intermittent preventive treatment, Artemether–lumefantrine

## Abstract

**Background:**

Malaria infections during pregnancy lead to sequestration of parasite infected red blood cells in the placenta. Placental infection can result in adverse outcomes for mothers and infants. Despite many studies, it remains unclear which peripheral blood infections during pregnancy lead to development of placental malaria. Understanding the timing of peripheral infections that lead to placental malaria and the ability of intermittent preventive treatment with sulfadoxine–pyrimethamine (SP-IPT) and artemisinin-based combination therapy to clear infections will enable the rational design of new interventions to decrease the burden of malaria in pregnancy.

**Methods:**

Microsatellite markers were used to genotype peripheral and placental malaria infections in an observational cohort in Blantyre, Malawi. Genotypes were compared to determine the timing of infections that sequester in the placenta. The effects of SP-IPT and artemether–lumefantrine as curative treatment were also evaluated by assessing the occurrence of peripheral infections or matching genotypes between peripheral and placental parasites following treatment.

**Results:**

Genotypes from 92 peripheral samples prior to delivery, 26 peripheral samples at delivery, and 29 placental samples were compared. Thirty percent of women with genotyped parasites in their placentas that had peripheral infections detected during pregnancy had matching peripheral-placental genotypes. Matching genotypes were not associated with gestational age and occurred from 13 to 39 weeks. Among women with more than one genotyped peripheral infection during pregnancy, 80 % had persistent infection with the same genotype while the remaining were new infections. Among infections treated with SP or artemether–lumefantrine, 28/84 (33 %) and 9/56 (16 %) had infection detected after treatment, respectively. Recrudescent infections were detected after both treatments and occurred up to 76 days after treatment. Women treated with SP-IPT and artemether–lumefantrine had genotypes matching treated infections detected in the placenta.

**Conclusions:**

Placental malaria can occur at any time during pregnancy. In the context of late enrollment in antenatal care, interventions that protect all women of childbearing age and throughout pregnancy are needed. Currently used medications do not always clear peripheral or placental infections. The ability of anti-malarial drugs to prevent or clear placental infections should be considered in the development of future interventions.

## Background

Malaria infection during pregnancy is a major public health concern in sub-Saharan Africa, where it is estimated to result in 100,000 infant deaths [1]. The adverse effects of malaria during pregnancy are due to sequestration of parasite-infected red blood cells in the placenta. Placental sequestration is mediated by parasite expression of a pregnancy specific variant surface antigen (VAR2CSA) on the red blood cell surface. Parasites from infections in all trimesters express VAR2CSA and are capable of binding to the placental target chondroitin sulfate A [2]. Women, especially in their first and second pregnancies, are particularly vulnerable because they have not acquired immunity to parasites expressing VAR2CSA [3]. Placental malaria infection is estimated to cause 900,000 low birth weight deliveries annually in sub-Saharan Africa through both intrauterine growth retardation and preterm delivery [4].

Previous studies have compared parasites detected at the time of delivery in the peripheral or cord blood to parasites detected in the placenta. While some studies have suggested that only a subset of peripheral infections sequester in the placenta, others have shown a large overlap between parasites found in each of these compartments [5–9]. Only one prior study has compared the parasite genotypes found in the maternal peripheral blood throughout pregnancy to the genotypes detected in the placenta. In that study, which was conducted in an area of seasonal, low malaria transmission, half of the women had placental genotypes detected in the periphery at some point prior to delivery and half of women had novel genotype(s) in the placenta. Treatment did not decrease the likelihood of finding matching genotypes in the periphery and in the placenta, suggesting that treatment may not always clear parasites from the periphery or the placenta [10].

Current recommendations to prevent malaria during pregnancy include intermittent preventive treatment with sulfadoxine–pyrimethamine (SP-IPT) after the first trimester, use of insecticide treated bed nets, and prompt and effective case management. The efficacy of these interventions is limited by behavioural factors, such as the timing of enrollment in antenatal care, and biological factors, such as the development of anti-malarial drug resistance. Women in sub-Saharan Africa often enroll in antenatal care and initiate SP-IPT late in the second or early in the third trimester when infection may have already occurred. Additionally, the spread of resistance to SP, especially in East Africa, threatens the efficacy of this strategy. Determining the timing of peripheral infections that lead to placental sequestration and the ability of treatment during pregnancy to clear the placenta could inform the development of more effective interventions decrease the burden of malaria in pregancy.

An observational cohort study in Blantyre, Malawi provided an opportunity to conduct a longitudinal examination of peripheral infections during pregnancy and compare them to placental infections. Parasite genotypes in the periphery detected throughout pregnancy were compared to each other and to genotypes found in the placenta in order to describe parasite dynamics during pregnancy. Determining the timing of peripheral infection leading to placental sequestration is challenging. In this study the timing of potential placental infection is inferred based on the gestational age at the first peripheral occurrence of a genotype detected in the placenta. While this method may be indirect, direct evidence of timing is limited by the fact that it is not feasible to sample the placenta at the time of peripheral infection prior to delivery. Parasites may not sequester immediately, but may subsequently alter their *var* gene expression and become capable of adhesion. Regardless of exact timing of sequestration, understanding the timing of infection with the potential for sequestration is critical to tailoring interventions. Because not all parasites are likely to express VAR2CSA, the hypothesis of this study is that only a subset of peripheral parasites would sequester in the placenta. Similarly, because women may encounter this subset of parasites at any time, peripheral infections at any time throughout pregnancy could lead to placental infection. This study also assessed the effects of SP as IPT and artemether–lumefantrine as curative treatment on peripheral infection. The effectiveness of these treatments in clearing the placenta was inferred by comparing the genotypes of parasites found at the time of treatment to those found in the placenta.

## Methods

### Study population

Four-hundred and fifty pregnant women were enrolled in an observational cohort study of malaria during pregnancy in Blantyre, Malawi between June 2009 and June 2010. All women were in their first or second pregnancy and were less than or equal to 28 weeks gestational age based on clinical assessment at enrollment. Women were followed monthly during pregnancy and encouraged to come to the clinic if they had intercurrent illness. At each encounter, peripheral blood smears and dried blood spots on filter paper were collected. At delivery, these same specimens and placental blood and tissue samples were collected. After quickening, women received SP-IPT up to three times separated by at least 4 weeks. Women with malaria detectable by blood smear at routine or sick visits were treated for malaria in accordance with the national guidelines: quinine in the first trimester and artemether–lumefantrine (AL) in the second and third trimesters. Early in the study a few smear positive women received SP-IPT as scheduled because they were asymptomatic. Molecular detection of infection was done after the study was complete and was not available for clinical care of the study participants. Details of the study design and results have been described previously [11]. Written informed consent was obtained from all participants. The study protocol and informed consent document were approved by the University of Malawi College of Medicine Research and Ethics Committee and the University of Maryland Institutional Review Board.

### Laboratory procedures

Procedures for specimen collection, placental biopsies, and microscopy were performed as described in Kalilani-Phiri et al. [11].

### Molecular detection

DNA was extracted from frozen placental samples and dried blood spots of peripheral blood and placental blood using the Qiagen QIAamp® 96 DNA Blood kit (Valencia, CA, USA). Quantitative real time polymerase chain reaction (qPCR) was used to detect the gene for *Plasmodium falciparum* lactate dehydrogenase. PCR master mix, primers and probes were purchased from Applied Biosystems (Foster City, CA, USA). Twenty-five microlitre reactions containing 1 μl extracted DNA were run. Extraction and qPCR protocols are described in detail on the University of Maryland website [12].

### Placenta evaluation

Placental malaria was defined as the presence of haemozoin pigment or parasites by histology or molecular evidence of infection using qPCR. Peripheral blood infections were categorized as either microscopic (smear positive, confirmed by qPCR) or submicroscopic (smear negative, but qPCR positive).

### Genotyping

All placental samples with parasites detected by qPCR underwent genotyping. Peripheral samples from any woman who had either placental infection and ≥1 peripheral infection or no placental infection and ≥2 peripheral infections were genotyped. Genotypes were based on six of the twelve unlinked microsatellite markers described in Anderson et al. [13]. Prior work demonstrated that these markers (Polyα, PfPK2, TA81, Ara2, TA87, and TA40) were sufficient to describe genetic diversity and relatedness among parasites in this specific population [14]. Product sizes of hemi-nested PCR reactions were resolved by capillary electrophoresis. Output files were analysed using Genemapper 4.0 (Applied Biosystems, Foster City CA, USA) with default microsatellite analyses settings. Genomic control strains: 3D7, HB3, and V1S (ATCC-MR4, Manassas VA, USA) were included to determine the characteristic morphology of peaks for each marker and to control for slight variations in capillary electrophoresis. Only peaks with the characteristic morphology above the threshold of two times the relative fluorescent units of background peaks in negative controls were scored. Fragment size was determined by manual inspection of each electropherogram and normalized against the 3D7 control. The adjusted peak sizes were rounded to the nearest nucleotide repeat size using a custom Perl script. Samples were analysed once the data had been adjusted and binned. Primers used in this study, as well as, microsatellite genotyping and scoring protocols are described in detail on the University of Maryland website [12].

### Data analysis

Samples with three or more detected microsatellite markers were considered evaluable. Genotypes were considered to match if identical alleles were detected in at least 4/6, 3/5, 3/4, or 2/3 of loci. The absence of matching of one allele at some loci was allowed because, with polyclonal infections, alleles may occasionally be undetectable [15]. Multiplicity of infection (MOI) was defined as the highest number of alleles found at a single locus in an infection and represents the number of simultaneous infections carried in a patient. Because of small numbers of peripheral samples from the time of delivery with infection, MOI was calculated with all peripheral samples prior to and at delivery.

Peripheral infections through pregnancy were classified as chronic, if matching genotypes were detected and new, if genotypes did not match. When assessing the ability of treatment to clear infections, infections were classified as recrudescent, if matching genotypes were detected, or new, if genotypes did not match. Gestational age at enrollment was calculated based on the last menstrual period or by the fundal height if the last menstrual period was not known.

Data analysis was performed using STATA version 12.1 software (Stata Corp, College Station, TX, USA). Student’s t-tests or Wilcoxon rank-sum were used for comparisons of normal and non-normal distributions of continuous variables, respectively. Chi-squared and Fisher’s exact tests were used for comparisons of proportions. For time to infection following treatment analysis, the log-rank test was used for univariate, categorical predictors and Cox proportional hazard regression was used for univariate analysis of continuous predictors and multivariable models. Predictors with p < 0.2 were included in model and interactions were evaluated. Statistical significance was set at p < 0.05.

## Results

All available peripheral blood (N = 2681) and placental (N = 317) samples from the 450 women enrolled in the study were screened for molecular evidence of infection [16]. Among the placental samples, 72 had molecular evidence of infection and evaluable genotypes were obtained from 29 (40 %). Genotyping was more likely to be successful in placentas with active infection detected by histology compared to placental parasites detected only by PCR (p = 0.008). Obtaining evaluable genotypes was also associated with higher concentrations of parasite DNA. Sixty-six percent of samples with DNA concentrations above the median able to be genotyped but only 24 % of samples equal to or below the median (p = 0.001). No evidence of a PCR inhibitor was detected.

One hundred ten peripheral samples from prior to delivery were genotyped either from women with evaluable placental genotypes or from women with more than one peripheral infection detected during pregnancy. Evaluable genotypes were obtained from 92 (84 %) peripheral infections.

Peripheral samples from the time of delivery were also genotyped if a placental genotype was available. Three women with placental genotypes available did not have a peripheral sample from delivery. Seven (27 %) of the 26 available samples had infection detected and all were able to be genotyped.

### Lower multiplicity of infection in the placenta compared to the periphery

Seventy-seven percent of peripheral infections were polyclonal. The mean MOI of peripheral infection was 3.5 (range 1–10). Two-thirds of placentas had polyclonal infections. The mean placental MOI was significantly lower than the MOI of peripheral samples [2.1 (range 1–4) p < 0.001]. There is no change in these results if peripheral samples from the time of delivery are removed from the analysis.

### Timing of peripheral infections associated with placental sequestration

**Fig. 1:**
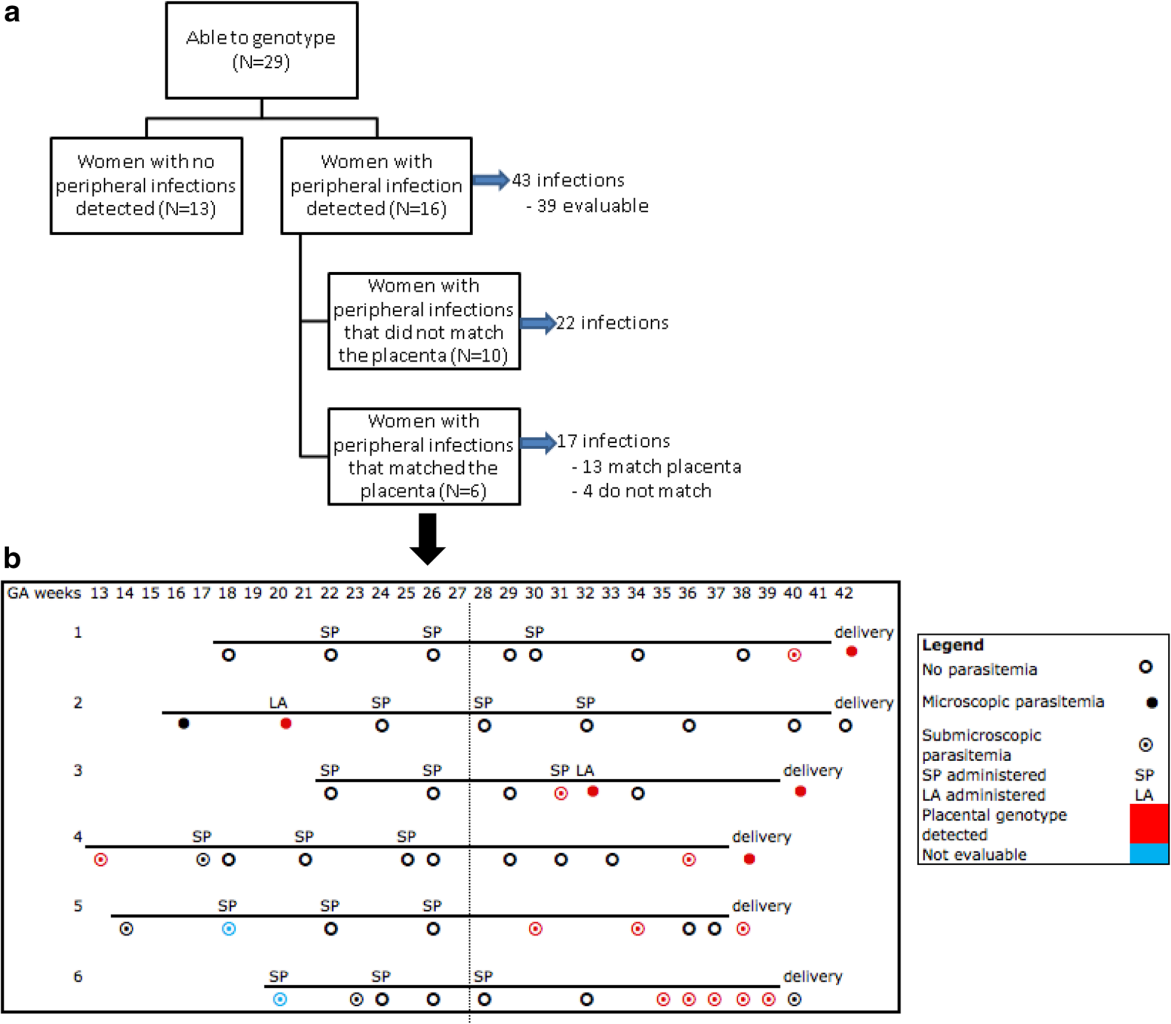
Comparison of placental and peripheral parasite genotypes. **a** Flow diagram of peripheral infections in women whose placentas were genotyped. **b** Infection and treatment history of women with matching peripheral and placental genotypes

Timing of peripheral infections associated with the presence of placental sequestration was assessed by matching parasite genotypes between peripheral infections placenta. Among 29 women with genotyped placental infections, sixteen (55 %) had at least one peripheral infection detected during pregnancy. These 16 women had 43 episodes of infection (Fig. 1a). One-third of women (6/16, 38 %) had placental parasite genotypes detected in the periphery prior to delivery that were identical to the parasites found in the placenta. Initial detection of a placental genotype in the periphery occurred at a range of gestational ages (13, 17, 27, 28, 33, and 39 weeks) (Fig. 1b). Five out of six of these women had placental genotypes present in the periphery in multiple specimens.

### Peripheral infections detected at delivery are more likely to share a placental genotype than peripheral infections earlier in pregnancy

Among women with genotyped placentas, the proportion of peripheral samples with infection prior to and at delivery was similar (20 vs 27 %, p = 0.44). At delivery, peripheral infections commonly matched the placental infection (5/7, 71 %). Whereas, less than one-third (13/43, 30 %) of peripheral infections prior to delivery contained the placental genotype (p = 0.08).

### Dynamics of infections in the periphery during pregnancy

**Fig. 2:**
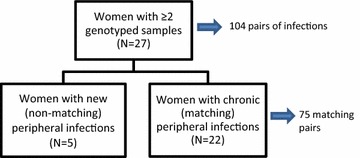
Flow diagram of peripheral infections in women with at least two infections during pregnancy

Twenty-seven women had evaluable genotypes from at least two peripheral infections during pregnancy. Twenty-two women (81 %) had at least one pair of matching peripheral genotypes (Fig. 2). Among women with more than one genotyped peripheral infection during pregnancy: 22 had chronic infections and five had new infections. Women with chronic infections were not different from women with new infections in terms of age, gravidity, gestational age at enrollment, number of visits, or placental malaria status (Table 1).

**Table 1:** Characteristics of women with chronic infections compared to new infections during pregnancy

	Chronic infection (N = 22)	New infection (N = 5)	p value
Mean age in years (95 % CI)	19.0 (17.7–20.3)	20.2 (16.4–24.0)	0.5
Primigravid (%)	82	60	0.3
Mean gestational age in weeks at enrollment (95 % CI)	18 (16–20)	16 (11–22)	0.5
Mean number of visits (95 % CI)	7.8 (6.7–8.9)	7.0 (4.0–10.0)	0.5
Had any placental malaria (%)	100^a^	75	0.2

^a^Among those with placental data available

When pairs of infections were compared, the initial infection of the pair occurred at later gestational ages when the subsequent infection was chronic as compared to new (23 vs 19 weeks, p = 0.05). There was no difference between the duration of time between the two samples. Administration of SP-IPT or AL between the two infections was not associated with having chronic or new infection (Table 2).

**Table 2:** Characteristics of chronic or new infections during pregnancy

	Chronic infection (N = 75)	New infection (N = 29)	p value
Gestational age in weeks at initial infection detection (95 % CI)	23 (19–28)	19 (15–24)	0.05
Days between samples (95 % CI)	46 (28–63)	74 (50–98)	0.08
SP-IPT between samples administered (%)	28	17	0.3
Artemether–lumefantrine between samples (%)	13	3	0.2

### Effects of SP-IPT and artemether–lumefantrine on parasite clearance from the periphery and the placenta

**Fig. 3:**
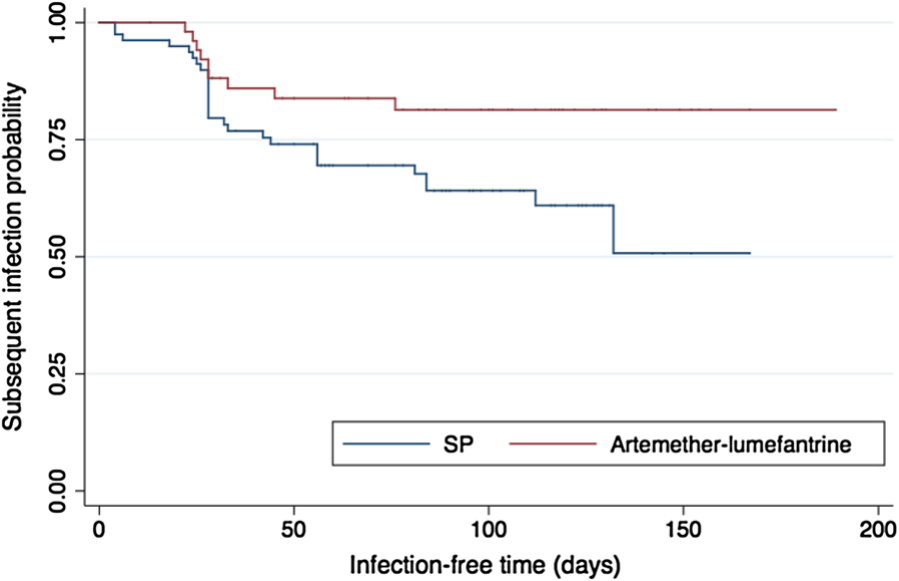
Peripheral infection following treatment with SP-IPT or artemether–lumefantrine. Kaplan–Meier curve of time to subsequent infection by treatment. Patterns of peripheral infection following treatment

Among infections for which women received SP-IPT or treatment with AL, 28/84 (33 %) and 9/56 (16 %) had subsequent infections, respectively. Treatment with AL decreased the rate of subsequent infection by 55 % when compared to SP (CI 5–80 %, p = 0.03) (Fig. 3). Gestational age at the time of initial infection, bed net use at the time of initial infection, and placental malaria were not associated with subsequent infection. Recrudescent infections were detected following both medications (Table 3). Recrudescence occurred up to 76 days after treatment.

**Table 3:** Ability of SP-IPT and AL to clear infections during pregnancy

Infection treated with	SP-IPT (N = 84)	AL (N = 56)
Any subsequent infection	28 (33)	9 (16)
Recrudescent infection (%)	13 (15)	7 (13)
New infection (%)	5 (6)	1 (2)
Type not able to be determined (%)	10 (12)	1 (2)

Figure 1b depicts the peripheral and placental genotypes from women treated with SP only or AL in addition to SP. Both groups have genotypes from treated infections detected in the placenta. For example, subject #4 received 3 doses of SP and no other anti-malarial medication. She had peripheral infection at 13 and 36 weeks gestational age with parasites of the same genotype as was detected in her placenta. Subjects #2 and #3 had microscopically detected infections that were treated with AL and matching genotypes from these peripheral infections were detected in their placentas. This implies that neither SP nor AL cleared the placenta of these genotypes or, alternatively, that neither drug cleared these genotypes from the periphery and they later sequestered in the placenta.

## Discussion

These data suggest there is no clear pattern of timing of peripheral infections resulting in placental infection, supporting the hypothesis that women are at risk of developing placental infection throughout pregnancy. Additionally, neither SP-IPT nor AL completely clears peripheral or placental infections. These findings have implications for the development of interventions to further decrease the burden of malaria during pregnancy.

Lower MOI in the placenta compared to peripheral samples and a 30 % concordance rate between parasites from the periphery and the placenta, suggest that not all peripheral infections lead to placental sequestration. While these results may be biased based on limitations of genotyping placental samples, the results have a strong pathophysiological basis. Placental sequestration of some peripheral infections may be limited because of either lack of variant surface antigen expression that can cause placental sequestration or the ability of AL or SP-IPT to clear some of these infections. Parasites infecting both the peripheral blood at delivery and the placenta have been shown to preferentially express VAR2CSA and in in vitro analyses have shown that parasites have the capacity to adhere to the placenta early in pregnancy [2]. Sequestration has also been shown to be influenced by maternal antibody responses to parasite pregnancy specific surface antigens [10]. This suggests that interventions that target parasites that sequester in the placenta, such as a VAR2CSA vaccine, may decrease placental infection, but may still allow for peripheral infection during pregnancy.

Women are vulnerable to placental infection throughout pregnancy. Thus, interventions that target a single period of pregnancy are unlikely to be successful in decreasing placental malaria. In this study, placental genotypes were found in the peripheral blood throughout pregnancy, without a consistent pattern. These data suggest that women acquire peripheral infections that eventually sequester in the placentas at all stages of pregnancy. More direct evidence could be provided by determining the VAR2CSA expression patterns and placental binding capability of initial peripheral infections. Future studies should consider these methods.

In the current study placental genotypes were more similar to peripheral genotypes at the time of delivery than to peripheral genotypes detected earlier in pregnancy. There are several possible explanations for the similarity of genotypes in the placenta and in the periphery at delivery: peripheral infection with parasites that sequestered in the placenta early in pregnancy have resolved by the time of delivery; new peripheral infections occurred late in gestation and infected erythrocytes sequestered in the placenta just before delivery; parasites previously sequestered in the placenta may be released into the periphery in response to the stress of labour; placental samples may be contaminated with maternal peripheral blood leading to an overrepresentation of matching genotypes between these two compartments. Regardless of the explanation, this highlights the importance of comparing infections from throughout pregnancy with placental infections at the time of delivery to further understand the dynamics of infection during this vulnerable period.

Of the six women with matching genotypes detected in the periphery and in the placenta, three had received SP only or artemether–lumefantrine in addition to SP suggesting that neither drug effectively clears either the periphery or the placenta. This is further supported by the finding that following treatment with either SP or AL, 14 % of women had recrudescence detected prior to delivery. While resistance to SP may explain the high rate of recrudescence following IPT, the high rate of recrudescence after treatment with AL is unanticipated. One explanation is that some anti-malarial drugs may not achieve therapeutic levels in the placenta. Thus, parasites sequestered in the placenta are protected and intermittently released into the peripheral blood. Parasites that evade clearance by sequestration in the placenta may have longer exposure to and thus greater chance of developing resistance to artemisinin partner drugs.

There are several important limitations to this study. Not all placental samples were successfully genotyped. Genotyping failures were due to low concentrations of parasite DNA in the placenta. It is unlikely that these technical challenges led to a bias in these results. Because of late enrollment in antenatal care, limited data from the first trimester is available. Infections present at enrollment, which are more common than after enrollment, likely represent the cumulative exposure during the first trimester and placental genotypes that do not match peripheral genotypes detected during follow-up may have been acquired prior to enrollment in antenatal care [11].

This study has several implications for the development of interventions to decrease the burden of malaria during pregnancy. If peripheral infections that lead to placental sequestration can occur at any time during pregnancy, then interventions to prevent or treat all infections during pregnancy are essential to protect the health of both mothers and their newborns. Interventions could target all women of childbearing age to prevent malaria infections during pregnancy. Anti-malarials that prevent or treat established infections in the placenta are needed. Given that neither SP-IPT nor AL appear to completely clear the placenta or the periphery of established infection, either new treatments that do clear the placenta or interventions to prevent all malaria infections during pregnancy are critical. Trials evaluating anti-malarial drugs for use during pregnancy need to monitor for recrudescence longer than the standard 28 or 42 days and include placental genotyping to evaluate the ability of drugs to clear the placenta.

## Conclusions

Placental malaria can occur at any time during pregnancy. Once infection is established in the placenta, currently available medications may not reliably clear the infection. In the context of late enrollment in antenatal care, interventions that protect all women of child bearing age and throughout pregnancy are needed. The ability of anti-malarial drugs to prevent or clear placental and peripheral infections should be considered in the development of future interventions.

## Abbreviations

AL: artemether–lumefantrine
MOI: multiplicity of infection
SP: sulfadoxine–pyrimethamine
SP-IPT: intermittent preventive treatment with sulfadoxine–pyrimethamine
qPCR: quantitative real time polymerase chain reaction
